# Nachhaltige Steigerung der Aktivität durch Rehabilitation

**DOI:** 10.1007/s00393-022-01179-4

**Published:** 2022-03-23

**Authors:** Monika Reuß-Borst, Johannes Boschmann, Fabian Borst

**Affiliations:** 1 Schwerpunktpraxis für Rheumatologie, Frankenstr. 36, 97708 Bad Bocklet, Deutschland; 2 Georg-August-Universität Göttingen, Göttingen, Deutschland; 3 Abteilung Orthopädie, Rehazentrum Bad Bocklet, Bad Bocklet, Deutschland

**Keywords:** Körperliche Aktivität, Rheuma, Muskuloskeletale Erkrankungen, Lebensstil, Reha-Dauer, Physical activity, Rheumatic diseases, Musculoskeletal disorders, Lifestyle, Rehabilitation duration

## Abstract

Patienten mit muskuloskeletalen Erkrankungen sind deutlich weniger körperlich aktiv als Gesunde. Sie können von einer Steigerung der körperlichen Aktivität und einer nachhaltigen Veränderung des Lebensstils in vielerlei Hinsicht profitieren, wozu eine stationäre Rehabilitation in erheblichem Maße beitragen kann. In dieser prospektiven Beobachtungsstudie (Prä-Post-Design) wurden körperliche Aktivität (mit dem Freiburger Fragebogen zur körperlichen Aktivität) und Depressivität (mit dem Beck-Depressions-Inventar [BDI]) bei 202 Rehabilitanden (124 weiblich, 77 männlich) mit muskuloskeletalen Erkrankungen (ICD-Diagnosen M) zu verschiedenen Katamnesezeitpunkten (zu Beginn der Reha, nach 3, 6, 9 und 12 Monaten) erhoben. Die Aktivitätssteigerung wurde in Abhängigkeit vom Ausgangslevel und der Depressivität analysiert. Drei Monate nach Rehabilitation lag das Aktivitätsniveau 47,8 % über dem Ausgangsniveau, was einer Steigerung der Medianaktivität von 5 auf 7,2 h pro Woche entspricht; 78,6 % der Teilnehmer zeigten nach 3 Monaten eine positive Differenz zum Ausgangsniveau. Der BDI-Score nahm im Mittel bei Durchführung der Rehabilitationsmaßnahme ab; eine Korrelation zwischen Abnahme des Scores und Zunahme der körperlichen Aktivität konnte nicht gezeigt werden. Durch eine 1‑malige Intervention (3-wöchige Rehabilitation) gelang eine Steigerung der körperlichen Aktivität über 12 Monate, wobei die Höhe der Aktivitätssteigerung nicht mit dem Ausgangslevel korreliert war, sodass auch bislang inaktive Patienten von der Reha profitierten.

## Abstract (en)

Patients with musculoskeletal diseases are much less physically active than healthy individuals. They could benefit from an increase in physical activity and a sustainable change in lifestyle in many ways, to which inpatient rehabilitation can make a substantial contribution. In this prospective observational study (pre-post design), physical activity (using the Freiburg physical activity questionnaire) and depressiveness (using the Beck depression inventory, BDI) were assessed in 202 rehabilitation patients (124 female, 77 male) with musculoskeletal disorders (ICD diagnoses M) at different catamnestic points in time (at the beginning of rehabilitation, after 3, 6, 9, and 12 months). The increase in activity was analyzed as a function of the activity level at the beginning as well as depressiveness. At 3 months after rehabilitation activity levels were 47.8% higher than at the beginning, corresponding to an increase in median activity from 5 to 7.2 h per week. Of the participants 78.6% showed a positive difference to the starting level after 3 months. The mean BDI score decreased during the rehabilitation intervention; a correlation between decreasing BDI and increasing physical activity could not be shown. A single intervention (3-week rehabilitation) succeeded in increasing physical activity over 12 months, whereby the increase in physical activity did not correlate with the initial activity level, concluding that even previously inactive patients benefit from rehabilitation.

Der westliche Lebensstil, insbesondere auch zunehmender Bewegungsmangel sind wichtige Ursachen für zahlreiche Zivilisationskrankheiten, wie z. B. Adipositas und assoziierte kardiovaskuläre und muskuloskeletale Erkrankungen. Die Weltgesundheitsorganisation (WHO) schätzt, dass 5,3 Mio. Todesfälle pro Jahr weltweit auf körperliche Inaktivität zurückzuführen sind [1]. WHO und American Society of Sports Medicine haben deshalb bereits vor Jahren klare Empfehlungen zur Häufigkeit und Intensität von körperlicher Aktivität publiziert, um gesundheitsförderliche Effekte für die Bevölkerung zu erreichen [2, 3]. Heute wird nicht nur gesunden Menschen, sondern auch Patienten mit degenerativen Gelenk- und Wirbelsäulenerkrankungen (Arthrosen, chronischen Rückenschmerzen), aber auch entzündlichen rheumatischen Erkrankungen eine Steigerung ihrer körperlichen Aktivität empfohlen. Viele Studien belegen, dass durch eine Steigerung der körperlichen Fitness der Operationszeitpunkt hinausgezögert, Lebensqualität gesteigert, Krankheitsaktivität reduziert und der Erhalt der Teilhabe (Partizipation) am beruflichen und sozialen Leben gesichert werden können [4, 5]. Bislang sind Patienten mit rheumatischen Erkrankungen meist deutlich weniger körperlich aktiv als Gesunde, wie zahlreiche Studien belegen konnten. Die Ursachen hierfür sind vielfältig [6–8].

Obwohl die Evidenzlage überzeugend ist, gelingt es vielen Patienten nicht, ihren oft über Jahrzehnte gepflegten (sedativen) Lebensstil zu ändern. Ärztliche Empfehlungen oder Initiativen von Landessportverbänden und Krankenkassen, wie z. B. ein „Rezept für mehr Bewegung“, führen meist nicht zu einer nachhaltigen Verhaltensänderung.

Hier könnte eine stationäre Reha-Maßnahme langfristig Erfolg versprechender sein; 40 % aller medizinischen Rehabilitationsmaßnahmen entfallen auf (chronische) muskuloskeletale Erkrankungen [9, 10]. Studien belegen, dass die meisten Patienten am Ende einer 3‑wöchigen Reha-Maßnahme körperlich aktiver sind und dies mit einer Verbesserung der Lebensqualität, Abnahme der Krankheitsaktivität und Schmerzintensität sowie weniger Depressivität und Fatigue einhergeht [11–14]. Die dauerhafte Umsetzung der während der Rehabilitation angestoßenen Verhaltensänderungen im häuslichen Umfeld gestaltet sich jedoch bei vielen Patienten erfahrungsgemäß schwierig. In dieser Studie adressierten wir die Frage, ob überhaupt eine langfristige Steigerung der körperlichen Aktivität durch Rehabilitation erreicht werden kann und ob es gelingt, auch bislang inaktive Patienten zu mobilisieren.

## Methodik

### Studiendesign

In dieser naturalistischen Beobachtungsstudie (Prä-Post-Design) wurden körperliche Aktivität und Depressivität bei 202 Rehabilitanden (124 weiblich, 77 männlich) im mittleren Alter von 52,8 Jahren mit muskuloskeletalen Erkrankungen (ICD-Diagnosen M) zu verschiedenen Katamnesezeitpunkten (Beginn der 3‑wöchigen Reha-Maßnahme, nach 3, 6, 9 und 12 Monaten) erfasst. Die Rekrutierung erfolgte von September 2019 bis September 2020. Die Studienteilnehmer wurden nach schriftlicher Einwilligung zu Beginn der Reha gebeten, den Freiburger Fragebogen zur körperlichen Aktivität sowie das Beck-Depressions-Inventar auszufüllen. Zu den verschiedenen Katamnesezeitpunkten wurden sie nochmals per Mail kontaktiert mit der Bitte, den Fragebogen elektronisch auszufüllen.

Beim Freiburger Fragebogen zur körperlichen Aktivität (FFkA) handelt es sich um einen validierten, in Studien häufig eingesetzten Fragebogen [15, 16], der Basisaktivitäten (z. B. Wege zu Fuß, mit dem Fahrrad, Treppensteigen), Freizeitaktivitäten (z. B. Tanzen, Kegeln, Spazierengehen) und sportliche Aktivitäten (z. B. Schwimmen und andere Sportarten) mit 12 Fragen erfasst, aus denen dann die gesundheitswirksame Gesamtaktivität (Stunden/Woche) errechnet werden kann.

Das Beck-Depressions-Inventar (BDI) ist ein in der Rehabilitation häufig eingesetztes validiertes, psychologisches Testverfahren, das die Schwere depressiver Symptome mit 21 Fragen erfasst [17]. Zur Auswertung werden alle Werte der einzelnen Aussagen addiert und dann mit Grenzwerten (Cut-off-Werten) verglichen. Ein höherer Score spiegelt eine stärkere Ausprägung depressiver Symptome wider.

### Auswertungsmethodik

Von 202 regelmäßig befragten Rehabilitanden liegen für 117 Personen zu allen Messzeitpunkten auswertbare Fragebögen vor. Dies entspricht einem kompletten Rücklauf über 12 Monate von 57,9 %. Grundsätzlich können einfache und multiple Imputationsverfahren (Regression, Last-Observation-Carried-Forward u. A.) angewendet werden, um unvollständige Datensätze zu korrigieren [18]. Insbesondere würde die oft verwendete Last-Observation-Carried-Forward-Imputation (LOCF) das Ergebnis in erheblichem Maße verfälschen, da eine unmittelbare Annahme (Aktivität zu einem nicht vorliegenden Zeitpunkt) über die eigentliche Zielgröße (zeitabhängiges Aktivitätsverhalten) getroffen wird. Aus diesem Grund werden im Folgenden ausschließlich über den kompletten Studienzeitraum vollständige Datensätze von 117 Patienten ausgewertet.

Zur Auswertung der Ergebnisse wurde folgende 4‑Schritt-Methodik angewendet. Im ersten Schritt wurde überprüft, ob die erfassten Ergebnisse statistische Signifikanz aufweisen. Hierfür wurde mithilfe des Kolmogorov-Smirnov-Tests analysiert, ob die erfassten Daten zu allen Zeitpunkten normalverteilt sind. Im Anschluss wurde die statistische Signifikanz für abhängige Stichproben mithilfe des Vorzeichentests überprüft. Für beide Tests wurde ein Signifikanzniveau α = 0,05 gewählt.

Nachdem die statistische Signifikanz nachgewiesen wurde, erfolgte im zweiten Schritt die übergeordnete Auswertung des Aktivitätsverhaltens der Grundgesamtheit zu verschiedenen Zeitpunkten. Hierbei wurde die absolute sowie relative Aktivitätsveränderung analysiert.

Im dritten Schritt wurde überprüft, welche Personengruppen eine besonders hohe Aktivitätssteigerung aufweisen und ob die Aktivitätssteigerung von der Anfangsaktivität abhängt. Hierfür wurden die Teilnehmer, basierend auf ihrer Anfangsaktivität, aufsteigend sortiert. Anschließend wurde der gleitende Mittelwert der Gesamtaktivität über 5 Personen zu Beginn der Studie und nach 3 Monaten verglichen. Die Berechnung des gleitenden Mittelwerts ${m}_{MA}^{n}$ für Probandennummer *j* erfolgte für 5 Personen ($n=5$) gemäß folgender Gleichung:

$${m}_{MA}^{n}\left(j\right)=\frac{1}{n}{\sum }_{i=0}^{n-1}\textit{Aktivit"at}\left(j-i\right)$$

Neben der Aktivitätsveränderung nach 3 Monaten wurde außerdem die Veränderung des Beck-Depressions-Inventars (BDI) in Abhängigkeit der Aktivitätsveränderung analysiert.

## Ergebnisse

Die Durchführung des Kolmogorov-Smirnov-Tests zeigte für ein Signifikanzniveau von 5 %, dass die Daten nicht normalverteilt sind. Aus diesem Grund wurde die statistische Signifikanz mit dem Vorzeichentest überprüft und nachgewiesen (Signifikanzniveau α = 0,05).

### Veränderung der Gesamtaktivität

**Figure 1:**
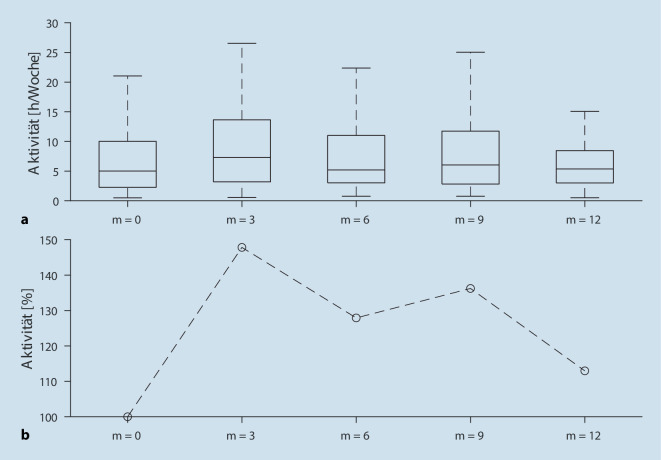

Die Abb. 1 zeigt die absolute und relative Veränderung der Gesamtaktivität zu verschiedenen Zeitpunkten. Hierbei ist erkennbar, dass der Median seinen Höhepunkt 3 Monate nach Beginn der Rehabilitationsmaßnahme mit einer relativen Aktivitätszunahme von 47,8 % erreicht. Im weiteren Verlauf nimmt die Gesamtaktivität ab, liegt aber nach 12 Monaten immer noch 13 % über dem Ausgangsniveau. Bei Betrachtung der absoluten Veränderung der Gesamtaktivität fällt auf, dass die Streubreite nach 3 und 9 Monaten höher als zu Beginn der Studie und nach 12 Monaten ist. Oberes und unteres Ende der Boxen visualisieren hierbei das 75. und 25. Perzentil.

### Veränderung der personenspezifischen Aktivität

**Figure 2:**
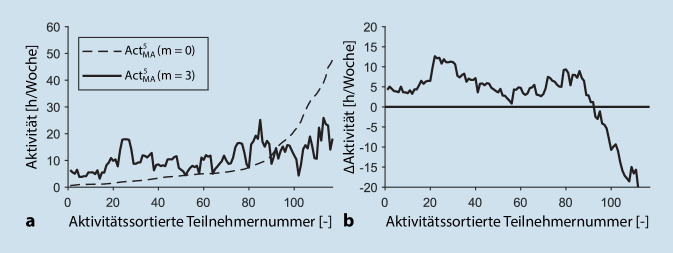

Die Abb. 2 stellt die Veränderung der Gesamtaktivität nach 3 Monaten in Abhängigkeit der personenspezifischen Anfangsaktivität dar. Hierfür werden die Probanden gemäß ihrer Anfangsaktivität (m = 0) aufsteigend sortiert, und die Aktivität nach 3 Monaten (m = 3) wird in Relation hierzu dargestellt. Zur Glättung wird, wie in Abschn. „Auswertungsmethodik“ beschrieben, der gleitende Mittelwert über 5 Personen berechnet. Anschließend wird die personenspezifische Aktivitätsdifferenz, die in Abb. 2b dargestellt ist, berechnet.

Hierbei zeigt sich, dass 78,6 % der Teilnehmer eine positive Differenz und damit eine Aktivitätssteigerung nach 3 Monaten verzeichnen. Die hohe Volatilität zeigt, dass die Höhe der Aktivitätssteigerung nicht maßgeblich mit der Anfangsaktivität korreliert ist, sondern von weiteren Faktoren abhängt. Auffallend ist außerdem, dass die Teilnehmer, die zu Beginn der Studie am aktivsten waren, sich nach 3 Monaten deutlich verschlechtert haben. Dies lässt vermuten, dass der Fragebogen fehlinterpretiert wurde (Aktivitätsstunden statt -minuten) oder einige Teilnehmer ihre Anfangsaktivität deutlich überschätzten.

### Veränderung des Beck-Depressions-Inventars

**Figure 3:**
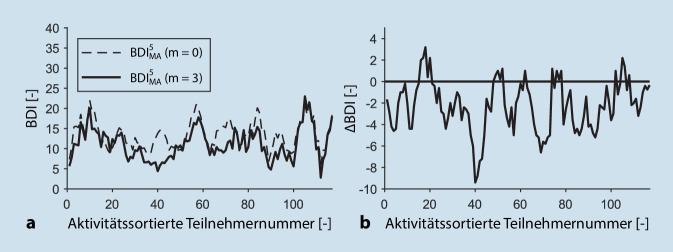

Die Abb. 3 zeigt die Veränderung der BDI-Werte nach 3 Monaten in Abhängigkeit der Aktivitätsveränderung. Hierbei wird die Teilnehmernummer gemäß der Aktivitätsveränderung nach 3 Monaten aufsteigend sortiert und der zugehörige BDI-Wert zu Beginn der Maßnahme sowie nach 3 Monaten dargestellt. In Abb. 3b wird zudem die BDI-Veränderung bei steigender Aktivitätsverbesserung visualisiert. Hierbei ist erkennbar, dass der mittlere BDI-Wert nach 3 Monaten um 17 % reduziert werden konnte. Eine Korrelation der BDI-Reduktion mit steigernder körperlicher Aktivität konnte nicht nachgewiesen werden, was die ähnlichen Veränderungen der BDI-Werte bei Teilnehmern mit geringer als auch hoher Aktivitätssteigerung visualisieren.

## Diskussion

Die körperliche Aktivität zu Reha-Beginn lag bei unserem Kollektiv mit 5,0 h/Woche deutlich niedriger als in der Studie von Frey et al. [15], die bei einer systematischen Stichprobe der Freiburger Bevölkerung (*n* = 612, Alter 20 bis 98 Jahre) eine mittlere Gesamtaktivität von 9,2 h/Woche ermittelten. Unser Ergebnis unterstreicht damit, dass Patienten mit muskuloskeletalen Erkrankungen zu Beginn der Rehabilitation deutlich weniger aktiv als gesunde Kontrollpersonen sind, was die Notwendigkeit gezielter Maßnahmen zur Steigerung der körperlichen Aktivität nochmals untermauert. Durch die multimodale Reha-Maßnahme konnte die körperliche Gesamtaktivität in unserem Kollektiv deutlich gesteigert werden. So wurde 3 Monate nach Abschluss der Rehabilitation eine relative Aktivitätszunahme von 47,8 % im Median erreicht. Im weiteren Verlauf nahm die Gesamtaktivität zwar wieder ab, lag aber nach 12 Monaten immer noch 13 % über dem Ausgangsniveau. Dies ist insofern bemerkenswert, da die Rekrutierung der Patienten während der Corona-Pandemie erfolgte und seit Beginn der Pandemie eine Abnahme der körperlichen Aktivität in der Bevölkerung festzustellen ist. Erfüllten vor der Pandemie noch 80,9 % der Bevölkerung die WHO(Weltgesundheitsorganisation)-Empfehlungen, waren einer aktuellen Studie in 14 Ländern zufolge dies zuletzt nur noch 62,5 % [19].

Eine 3‑wöchige Reha-Maßnahme führt damit nachweislich zu einer Steigerung der körperlichen Aktivität. Auffallend ist dabei die hohe Volatilität der Aktivitätssteigerung. Diese hohe Volatilität der Aktivitätssteigerung zwischen Personen mit ähnlicher Anfangsaktivität weist darauf hin, dass die Höhe der Aktivitätssteigerung von vielen Faktoren beeinflusst wird, die im Rahmen einer stationären Rehabilitation wirksam werden, wie z. B. auch unterschiedliche edukative Elemente (Patientenschulungen, motivationale Einheiten). Interessanterweise wurde sowohl bei primär inaktiven als auch bereits aktiven Patienten eine Zunahme der Gesamtaktivität beobachtet. Die Steigerung der körperlichen Aktivität ist damit nahezu unabhängig vom Ausgangslevel – auch bislang inaktive Patienten profitieren von einer Reha.

Durch die multimodale Reha-Maßnahme konnte die körperliche Gesamtaktivität deutlich gesteigert werden

Gerade für letztere Gruppe von Patienten kann die Rehabilitation ein wichtiger Anstoß für eine Verhaltensänderung sein und sollte deshalb auch von behandelnden Ärzten empfohlen werden. Eine Steigerung der körperlichen Aktivität geht oft auch mit einer Abnahme der Depressivität einher. Dies war in unserer Studie nicht der Fall. Eine Korrelation der BDI-Reduzierung mit steigender körperlicher Aktivität konnte nicht nachgewiesen werden, auch wenn körperliche Aktivität bei Depressionen durchaus therapeutisch wirken kann [20]. Dies führen wir darauf zurück, dass im Rahmen der multimodalen Reha-Maßnahme auch psychologische Einzel- und Gruppeninterventionen zum Einsatz kommen, die ebenfalls wirksam sind, sodass die Verbesserung des BDI durch die Rehabilitation von unterschiedlichen Interventionen abhängt.

Bislang ist das Antragsverfahren für eine stationäre oder ambulante Reha-Maßnahme komplex und für viele Patienten, aber auch Ärzte wenig transparent – ein Grund, weshalb die Zahl der Reha-Maßnahmen in den letzten Jahren sogar leicht rückläufig war [21]. Unsere Ergebnisse belegen, dass eine Rehabilitation nachhaltige Lebensstiländerungen anstoßen kann und auch Patienten profitieren, die bislang kaum körperlich aktiv waren. Um diesen Erfolg auch langfristig zu verstetigen, wäre eine Flexibilisierung von Reha-Dauer und auch Reha-Intervallen nach individuellen Bedürfnissen unserer Meinung nach sinnvoll. Bei onkologischen Rehabilitanden konnten wir bereits in einer Studie zeigen, dass durch eine individuelle Nachsorge mit 1‑wöchigen Etappenheilverfahren nach 4 und 8 Monaten eine signifikante Steigerung der körperlichen Aktivität sogar über 2 Jahre möglich ist [22].

Unsere Studie hat einige Schwächen, auf die ebenfalls hingewiesen werden soll. Es ist nicht auszuschließen, dass nur besonders motivierte Patienten an der Studie teilgenommen haben und auch nur von diesen ein kompletter Datensatz vorliegt. Allerdings weist die hohe Streuweite der Aktivität (s. Abb. 1) auf ein äußerst heterogenes Probandenspektrum hin. Darüber hinaus hat sich gezeigt, dass die Mehrdeutigkeit des Freiburger Fragebogens zur körperlichen Aktivität (wahlweise Angabe der Aktivität in Stunden bzw. Minuten) zu nicht eindeutigen Ergebnissen führt. Dies spiegelt sich auch darin wider, dass sich die Ergebnisse der anfangs aktivsten Patienten dem Niveau der übrigen Teilnehmer zum Zeitpunkt t1 annähern. Hier ist davon auszugehen, dass die Angabe zum Zeitpunkt t0 in Minuten und zum Zeitpunkt t1 in Stunden erfolgt ist. Durch den Einsatz von „wearables“ wie Schrittzählern wären hier sicher noch validere Ergebnisse zu erwarten.

Zusammengefasst zeigt unsere Studie, dass bei Patienten mit muskuloskeletalen Erkrankungen eine Steigerung der körperlichen Aktivität um 47 % (auch unter den Bedingungen einer Pandemie) möglich ist. Um den Lebensstil nachhaltig, d. h. über Jahre zu ändern, dürfte allerdings eine 1‑malige 3‑wöchige Intervention nicht ausreichen. Hier könnten zusätzliche ambulante und stationäre Angebote helfen, den Therapieerfolg langfristig zu verstetigen.

## Fazit für die Praxis

- Die körperliche Aktivität kann bei Patienten mit muskuloskeletalen Erkrankungen durch Rehabilitation nachhaltig gesteigert werden.
- Drei Monate nach Abschluss der Rehabilitation wurde eine relative Aktivitätszunahme von 47,8 % erreicht.
- Die Steigerung der körperlichen Aktivität war unabhängig vom Ausgangslevel – auch bislang inaktive Patienten profitieren von einer Rehabilitation.
- Zwölf Monate nach Beendigung der Reha-Maßnahme waren die Patienten noch aktiver als zu Reha-Beginn.
- Um diesen Erfolg auch langfristig zu verstetigen, erscheint eine Flexibilisierung von Reha-Dauer und auch Reha-Intervallen nach individueller Situation notwendig.
